# Appropriate use of antiplatelet medications following transient ischemic attacks and stroke: a 9-year study from the Middle East

**DOI:** 10.3389/fneur.2023.1269292

**Published:** 2023-11-09

**Authors:** Hiba Naveed, Naveed Akhtar, Salman Al-Jerdi, Ryan Ty Uy, Sujatha Joseph, Deborah Morgan, Blessy Babu, Shobana Shanthi, Ashfaq Shuaib

**Affiliations:** ^1^Department of Medical Education, Weill Cornell College of Medicine, Doha, Qatar; ^2^The Neuroscience Institute, Hamad Medical Corporation, Doha, Qatar; ^3^Neurology Division, Department of Medicine, University of Alberta, Edmonton, AB, Canada

**Keywords:** TIA, stroke, antiplatelet, modified Rankin scale, guidelines, DAPT

## Abstract

**Background and purpose:**

Guidelines recommend that patients with high-risk TIAs and minor strokes presenting within 1–3 days from onset should be offered dual antiplatelet therapy (DAPT). There are little data on real-world adherence to these recommendations. We evaluated the appropriateness of DAPT use in TIA and stroke patients in a prospective database.

**Methods:**

The Qatar Stroke Database began the enrollment of patients with TIAs and acute stroke in 2014 and currently has ~16,000 patients. For this study, we evaluated the rates of guideline-adherent use of antiplatelet treatment at the time of discharge in patients with TIAs and stroke. TIAs were considered high-risk with an ABCD2 score of 4, and a minor stroke was defined as an NIHSS of 3. Patient demographics, clinical features, risk factors, previous medications, imaging and laboratory investigations, final diagnosis, discharge medications, and discharge and 90-day modified Rankin Scale (mRS) were analyzed.

**Results:**

After excluding patients with ICH, mimics, and rare secondary causes, 8,082 patients were available for final analysis (TIAs: 1,357 and stroke: 6,725). In high-risk TIAs, 282 of 666 (42.3%) patients were discharged on DAPT. In patients with minor strokes, 1,207 of 3,572 (33.8%) patients were discharged on DAPT. DAPT was inappropriately offered to 238 of 691 (34.4%) low-risk TIAs and 809 of 3,153 (25.7%) non-minor stroke patients.

**Conclusion:**

This large database of prospectively collected patients with TIAs and stroke shows that, unfortunately, despite several guidelines, a large majority of patients with TIAs and stroke are receiving inappropriate antiplatelet treatment at discharge from the hospital. This requires urgent attention and further investigation.

## 1. Introduction

Early initiation of antithrombotic medications decreases the risk of recurrence of vascular events in patients with coronary artery disease (CAD), transient ischemic attack (TIA), and ischemic stroke (1–5). Numerous clinical trials during the last 50 years have provided evidence that ASA and clopidogrel reduce the risk of stroke and other vascular events when compared to placebo (6–13). The risk of stroke and other vascular diseases following TIA and stroke is highest in the initial days following the event (REF). The ABCD2 score was designed to improve the identification of patients at the highest risk of recurrent events (REFs). The score was further refined with the addition of imaging criteria (REF).

Several recent trials have shown that short-term combination antiplatelet treatment is superior to that of single antiplatelet agents (14–16). Two larger, more recent trials have shown that DAPT for 21–90 days initiated early following a high-risk TIA or minor stroke may result in a significant reduction of stroke recurrence (15, 16). In the CHANCE trial, DAPT was used for 21 days in patients presenting within 24 h from the onset of symptoms (15), whereas the POINT trial used DAPT for 90 days in patients with high-risk TIAs or minor strokes (NIHSS ≤ 3 on admission), initiated within 12 h from the onset of symptoms (16). The significantly better outcome with a combination of ASA and clopidogrel treatment resulted in strong “level A class I” in guideline statements from the United States (7, 8), Canada (17), and Europe (18).

The recommendations for the use of DAPT are specific for high-risk TIAs (ABCD2 score of ≥ 4) or minor strokes (NIHSS ≤ 3 on admission). However, there is very little information offered in the guidelines on the avoidance or inappropriate use of DAPT in low-risk TIAs or patients with moderate-to-large strokes (NIHSS of ≥ 4). Three recent publications reported the underutilization of DAPT in high-risk patients with minor strokes (19–21). The Get-With-The-Guidelines Stroke program, which evaluates stroke treatment in 1800 hospitals, reported that only 47% of patients with minor strokes were offered DAPT in 2019. The same study also showed that inappropriate DAPT was offered to 42.6% of patients with non-minor strokes (19). The second study, following the publication of the CHANCE and POINT studies (19, 20), revealed that appropriate use of DAPT remains low, especially in non-comprehensive stroke centers [comprehensive stroke centers (54.7%) vs. primary stroke centers (23.1%)] (20). The third study from Italy showed similar low use of DAPT in high-risk patients (21).

In this communication, we report on the antiplatelet treatment of 8,082 patients with TIAs (*n* = 1,357) and ischemic stroke (*n* = 6,725) who were prospectively entered into our stroke registry between 2013 and 2022. Our specific aims include the evaluation of factors that were associated with low rates of DAPT usage in patients with high-risk TIAs and minor strokes. An additional goal was to identify the reasons for the inappropriate use of DAPT in low-risk TIAs or patients with non-minor strokes.

## 2. Methods

This study comprised patients who were prospectively entered into the stroke program database. The study was approved by the Committee for Human Ethics Research, Academic Health Service at HMC (MRC-01-20-1135).

All patients admitted via the emergency department with a provisional diagnosis of stroke, including ischemic stroke, stroke mimics, transient ischemic attack (TIA), and intracranial hemorrhage (ICH), in Hamad General Hospital (HGH), Doha, Qatar, between 1 January 2014 and 4 December 2021, were entered into the database by four specially trained advanced nurse practitioners. For the purpose of this study, we only evaluated patients with a confirmed diagnosis of TIA and ischemic stroke. We excluded patients for whom the final diagnosis was either ICH or stroke mimics. The details of the stroke database have previously been published (22–26). In brief, HGH is a Joint Commission International-accredited 600-bed hospital, where ~95% of all strokes in Qatar requiring hospitalization are admitted. The stroke program, also certified by the Joint Commission International, is equipped with all the necessary laboratory, neuro-radiological, and neurosurgical facilities needed to manage acute stroke patients and has 24-h thrombolysis and thrombectomy services. The stroke service is run by seven stroke-trained neurologists and includes clinical pharmacists, nurse practitioners, physical and occupational therapists, and speech-language pathologists. All patients with a suspected diagnosis of acute stroke are evaluated in the emergency department (ED) by the stroke team to enable immediate decisions on acute stroke interventions and further management. The majority of patients are admitted to a designated stroke ward.

Patients or the public were not involved in designing, conducting, reporting, or disseminating the plans of our research.

### 2.1. Patient characteristics and data collection

The anonymized data entered into the HGH stroke database (Microsoft Office Access 2007 Database) include patient characteristics such as age, sex, nationality, medical comorbidities, and prior medication. We specifically evaluated the prior use of antithrombotic medications and whether the patients were taking any medications for common stroke risk factors, including antihypertensive, antidiabetic, and statin medications.

Upon identification in the ED, data were collected once confirmation of the diagnosis of ischemic stroke was made using the International Classification of Disease, 10th Edition, definitions (H34·1, I63.x, I64.x, I61.x, I60.x, G45.x). Data from emergency medical services/paramedics, immediate ED care, door-to-needle time (for thrombolysis patients), prior mRS, NIHSS score, length of stay (LOS), neuroimaging, post-stroke complications, in-hospital mortality, and recurrences were recorded. The modified Rankin scale (mRS) measurements were done at discharge and at 90 days (27). Patients were classified as having a good (mRS of ≤0–2) or poor (mRS of 3–6) outcome. We used the TOAST classification (28) for the final diagnosis of stroke etiology. This classification defines patients in the following categories with predefined criteria: large vessel disease, small vessel or lacunar stroke (SVD), cardioembolic, and stroke of determined or undetermined etiology (28).

Hypertension was diagnosed with blood pressure higher than 140 mmHg systolic and 90 mmHg diastolic, as defined by the International Society of Hypertension (29). Dyslipidemia was defined as a low-density lipoprotein-cholesterol (LDL) level of ≥ 3.62 mmol/L, high-density lipoprotein-cholesterol (HDL) level of ≤ 1.03 mmol/L, triglyceride level of ≥ 1.69 mmol/L, or current treatment with a cholesterol-lowering drug (30). Atrial fibrillation (AF) was diagnosed based on electrocardiographic findings on admission or Holter monitoring during hospitalization. Smoking was defined as current cigarette smoking. Complications monitored and recorded included aspiration pneumonia, urinary tract infection, bedsores, and sepsis during hospitalization. Diabetes was diagnosed according to the American Diabetes Association (ADA) and WHO recommendations and included patients with a previous diagnosis of DM, on medication for DM or an HbA1c ≥6.5%, and the diagnosis of pre-DM was based on an HbA1c of 5.7–6.4 % as per 2015 ADA clinical practice recommendations (31).

For this study, we specifically evaluated the timing of the initiation of antiplatelet treatment in patients with non-cardioembolic TIAs and ischemic strokes. The prior use of any antiplatelets was documented. We also documented the time from the initial diagnosis of TIA, or ischemic stroke, to the initiation of SAPT or DAPT treatment in the hospital for all patients. The database records the patient outcome at 90 days (mRS) and 1-year follow-up of major cardiovascular events (MACEs), but unfortunately does not document if the patient develops any complications (specifically intracranial or systemic bleeding). We are, therefore, unable to determine bleeding-related complications in the patients listed in the database. We also did not record if the patient had a prior history of systemic or intracranial hemorrhage.

To collect post-discharge, follow-up data, the Cerner electronic medical systems were used to track patient admissions throughout the state of Qatar. We coded patient outcomes using major MACE scores based on data in the Cerner files. We collected data on recurrent stroke, post-stroke myocardial infarction (non-fatal and fatal), cardiac arrest, post-stroke cardiac revascularization, and death for 1 year. We also conducted separate analyses on patients with high-risk TIAs and low-risk TIAs and on patients with minor and non-minor stroke patients, comparing their discharged status on appropriate and inappropriate DAPT, as shown in Supplementary Tables 1, 2.

### 2.2. Appropriate use of antiplatelet agents

**Figure 1:**
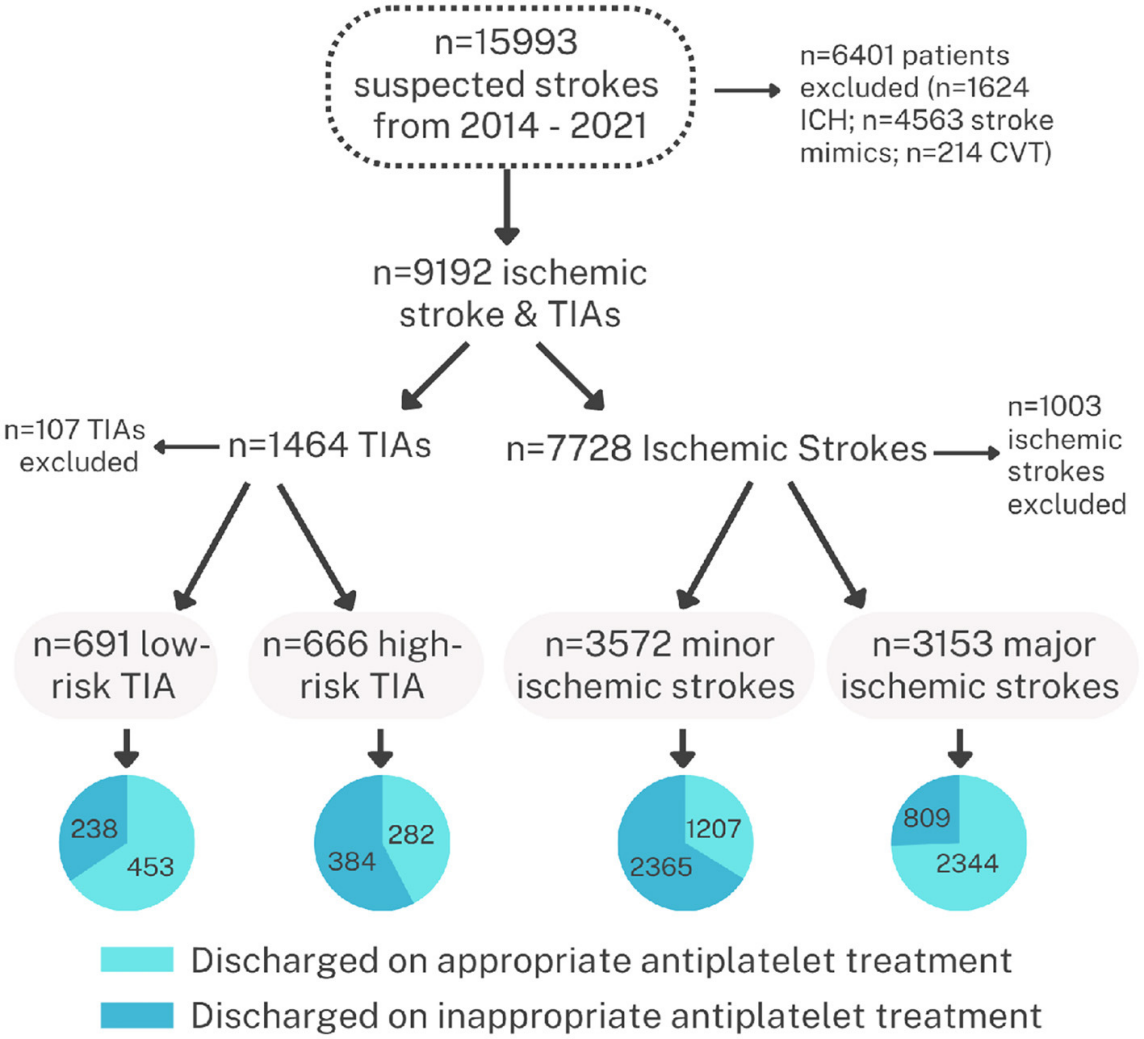
Flow diagram of the patients in the database used for the current analysis showing the total number of patients enrolled in the registry and the treatment at discharge in patients with TIAs and stroke.

The main objective of the current study was the appropriate use of antiplatelet agents in patients presenting with a diagnosis of TIA or acute stroke. We used the American Heart Association/American Stroke Association recommendation for DAPT use (21–90 days) in patients with high-risk TIA and minor stroke. Patients with TIAs within 3 days of an ABCD2 score of ≥ 4 and NIHSS ≤ 3 on admission were considered appropriate for DAPT (8). We, therefore, analyzed the data in four major categories, as shown in Table 1. Patients with TIAs (*n* = 1,357) were classified into high-risk (*n* = 666) and low-risk (691) TIAs. The appropriate use of DAPT and SAPT was evaluated in this group. We considered DAPT appropriate only in patients with an ABCD2 score of ≥ 4 in whom treatment could be initiated within 3 days from the symptom onset. DAPT in all other TIA patients was considered inappropriate (Figure 1). For patients with completed strokes, we considered the use of DAPT “appropriate” when treatment was initiated within 3 days in patients with NIHSS ≤ 3. In all other patients with stroke, the use of DAPT was considered “inappropriate.”

**Table 1:** Baseline characteristics of patients admitted with TIA and discharged on appropriate antiplatelet therapy.

**Characteristic or investigation**	**Total high-risk TIAs (*n* = 666)**	**Discharged on inappropriate therapy (*n* = 384, 57.7%)**	**Discharged on appropriate therapy (*n* = 282, 42.3%)**	***P*-value**	**Total low-risk TIAs (*n* = 691)**	**Discharged on Inappropriate therapy (*n* = 238, 34.4%)**	**Discharged on appropriate therapy (453, 65.6%)**	***P*-value**
Age, mean, years	56.1 ± 13.2	55.78 ± 13.7	56.5 ± 12.5	0.460	51.05 ± 11.8	51.5 ± 11.4	50.82 ± 12.0	0.47
Sex male	481 (72.0)	247 (64.3)	234 (83)	< 0.001	546 (79.0)	197 (82.8)	349 (77)	0.08
Hypertension	485 (72.8)	285 (58.8)	200 (41.2)	0.34	401 (58)	155 (38.7)	246 (61.3)	0.006
Diabetes	403 (60.5)	241 (59.8)	162 (40.2)	0.16	253 (36.6)	95 (37.5)	158 (62.5)	0.19
Dyslipidemia	340 (51.1)	203 (59.7)	137 (40.3)	0.27	285 (41.2)	101 (35.4)	184 (64.6)	0.64
Prior stroke	87 (13.1)	50 (57.5)	37 (42.5)	0.97	64 (9.3)	27 (42.2)	37 (57.8)	0.17
Atrial fibrillation	15 (2.3)	9 (60.0)	6 (40.0)	0.85	12 (1.7)	2 (16.7)	10 (83.3)	0.19
Coronary artery disease	89 (13.4)	43 (48.3)	46 (51.7)	0.05	70 (10.1)	34 (48.6)	36 (51.4)	0.009
Active smoking	151 (22.7)	77 (51.1)	74 (49.0)	0.06	197 (28.5)	64 (32.5)	133 (67.5)	0.49
Obesity (BMI ≥30 kg/m^2^)	233 (35.9)	147 (63.1)	86 (36.9)	0.04	213 (31.8)	77 (36.2)	136 (63.8)	0.65
**Ethnicity**								
Arabs	305 (45.8)	198 (64.9)	107 (35.1)	0.01	263 (38.1)	91 (34.6)	172 (65.4)	0.40
South Asian	254 (38.1)	128 (50.4)	126 (49.6)		289 (41.8)	108 (37.4)	181 (62.6)	
Far Eastern	50 (7.5)	27 (54.0)	23 (46.0)		69 (10)	21 (30.4)	48 (69.6)	
African	37 (5.6)	19 (51.4)	18 (48.6)		33 (4.8)	9 (27.3)	24 (72.7)	
Caucasian	20 (3.0)	12 (60.0)	8 (40.0)		37 (5.4)	9 (24.3)	28 (75.7)	
**Prior antiplatelet use**								
None	438 (65.8)	255 (58.2)	183 (41.8)	0.68	535 (77.4)	173 (32.3)	362 (67.7)	0.03
Antiplatelet use	228 (34.2)	129 (56.6)	99 (43.4)		156 (22.6)	65 (41.7)	91 (58.3)	
Prior anti-hypertensive use	259 (38.9)	155 (59.8)	104 (40.2)	0.36	213 (30.8)	78 (36.6)	135 (63.4)	0.42
Anti-diabetic medication use	223 (33.5)	139 (62.3)	84 (37.7)	0.08	144 (20.8)	53 (36.8)	91 (63.2)	0.50
Prior statin use	227 (34.1)	134 (59.0)	93 (41.0)	0.61	168 (24.3)	68 (40.5)	100 (59.5)	0.06
Initiation of antiplatelet within 24 h	465 (69.8)	261 (56.1)	204 (43.9)	0.22	450 (65.1)	168 (37.3)	282 (62.7)	0.03
Mean duration to start antiplatelet (h)	10.6 ± 11.5	11.02 ± 12.8	10.13 ± 9.5	0.32	12.01 ± 13.0	10.94 ± 9.9	12.58 ± 14.3	0.12
**Antiplatelet given after the onset**								
< 24 h	465 (69.8)	261 (56.1)	204 (43.9)	0.28	450 (65.1)	168 (37.3)	282 (62.7)	0.08
Between 24 and 48 h	133 (20)	78 (58.6)	55 (41.4)		152 (22)	48 (31.6)	104 (68.4)	
Between 48 and 72 h	47 (7.1)	29 (61.7)	18 (38.3)		52 (7.5)	15 (28.8)	37 (71.2)	
>72 h	21 (3.2)	16 (76.2)	5 (23.8)		37 (5.4)	7 (18.9)	30 (81.1)	
Mean ABCD2 score	4.7 ± 0.8	4.69 ± 0.8	4.71 ± 0.8	0.82	1.68 ± 1.29	1.75 ± 1.31	1.65 ± 1.28	0.33
**ABCD2 score at presentation**								
0–1					262 (37.9)	83 (31.7)	179 (68.3)	0.23
2–3					429 (62.1)	155 (36.1)	274 (63.9)	
4–5	554 (83.2)	317 (57.2)	237 (42.8)	0.61				
6–7	112 (16.8)	67 (59.8)	45 (40.2)					

DUAP, dual antiplatelet therapy; IV, intravenous; NIHSS, National Institute of Health Stroke Scale; RBS, random blood sugar; HDL, high-density lipoprotein; LDL, low-density lipoprotein; BMI, body mass index; mRS, Modified Rankin score; CABG, coronary artery bypass graft; PCI. percutaneous coronary intervention; MACE, major cardiac adverse event.

The independent sample t-test was applied to test for significant differences across antiplatelet use for all continuous variables, whereas the chi-square test was used for all categorical variables.

Antiplatelet treatment was categorized into the following groups: ASA alone, clopidogrel alone, ASA+clopidogrel, ASA+dipyridamole, and ASA+other antiplatelet agents such as ticlopidine, prasugrel, cilostazol, or ticagrelor. Although DAPT may include any two antiplatelet agents, the combination of ASA+clopidogrel was the only one used for the patients listed in the database.

### 2.3. Data analysis and statistics

Descriptive results for all quantitative variables (e.g., age, systolic BP, BMI, and others, as shown in Table 1) were reported as mean ± standard deviation (SD). Numbers (%) were reported for all categorical variables (e.g., gender, diabetes, atrial fibrillation, and others, as shown in Table 1). The distribution of continuous variables was assessed by applying the Kolmogorov–Smirnov test prior to using statistical tools.

The independent sample *t*-test was applied to test for significant differences across antiplatelet use for all continuous variables, whereas the chi-square test was used for all categorical variables. The independent sample *t*-test was used to compare the average for all the quantitative variables, and Pearson's chi-square test or Fisher's exact test was used to compare the proportion of all qualitative variables.

A univariate analysis using Pearson's chi-square test or Fisher's exact test showed short-term 90-day outcomes and major cardiovascular events (MACEs) in patients with TIA admitted and discharged on appropriate vs. inappropriate dual antiplatelet therapy (DAPT) and in patients with minor and non-minor strokes discharged on appropriate and inappropriate DAPT (Supplementary Tables 1, 2).

A *p*-value of 0.05 (two-tailed) was considered to be a statistically significant level. SPSS 21·0 statistical package was utilized for the analysis.

## 3. Results

During the study period (2014–2022), there were 15,993 patients prospectively enrolled in the database. We excluded patients with ICH (1,624), stroke mimics (4,563), cerebral venous sinus thrombosis (214), and patients that required long-term anticoagulation (640), leaving us with a total of 8,082 patients in whom discharge medications included single or dual antiplatelet medications. There were 1,357 patients with TIAs (including 666 patients with an ABCD2 score of ≥ 4) and 6,725 patients with ischemic stroke (including 3,572 patients with minor stroke [NIHSS ≤4]). The patient population was predominantly male expatriate workers reflecting the demographics of Qatar which has a large expatriate working population (http://www.mdps.gov.qa/english/population). Female patients were mostly Qatari locals or long-stay expatriates and were significantly older in age. The expatriate patient population was mostly Arabs (45.8%) or South Asians (38.1%).

### 3.1. Prior use of antiplatelet agents at the time of the TIA or stroke

Prior to the TIA or stroke, 6,291 (77.8%) of patients were *naive* to antiplatelet treatment. Aspirin was the most common antiplatelet agent (1,304, 16.1%) prior to the acute event. Clopidogrel was the SAPT in 149 (1.8%) patients. Of the 1,357 TIA patients, 322 (23.7%) were on SAPT, and only 62 (4.6%) were taking DAPT. There was no difference in the appropriate or inappropriate use of DAPT in relation to the prior use of antiplatelet therapy, as shown in Table 1. Similarly, the majority of patients with stroke were also not on any antiplatelet treatment prior to the acute event (5,318 of 6,725 [79.1%]). There were 1,131 patients (16.8%) on prior SAPT at the time of the stroke, including 17% with a minor stroke and 16.5% with a non-minor stroke.

### 3.2. Distribution of antiplatelet treatment in patients with TIAs

The distribution of antiplatelet treatment at the time of discharge for TIAs and minor strokes is shown in Table 1. In patients with high-risk TIAs, 282/666 (42.3%) were discharged on DAPT and 384/666 (57.7%) on SAPT. At the time of discharge, SAPT was offered to 453/691 [65.6%] of low-ABCD2 patients, and DAPT was offered to 238/691 (34.4%) of such patients. We next evaluated the relationship between increasing ABCD2 scores and the use of DAPT. As shown in Table 1, there appeared to be no difference in DAPT use with the ABCD2 score between 4 and 7. The frequency of DAPT use in patients with an ABCD2 score of 0–3 was marginally lower, especially in the ABCD2 with scores of 0–2. In our population, there is a higher frequency of symptoms related to the posterior circulation. There were 274/1,357 (20.2%) TIAs of posterior circulation origin, and 55% had an ABCD2 score of ≤3. The recommended treatment in such patients is SAPT, as the CHANCE (15) and POINT (16) trials excluded these patients, and the most current national guidelines (7, 8, 17, 18) do not recommend DAPT in such patients.

We next evaluated if patients presenting within 24 h of onset were more likely to be offered DAPT. As shown in Table 1, time to treatment from onset seems to make no difference in treatment. The rates of DAPT were similar in patients presenting early (within 24 h) or late (more than 72 h).

We also evaluate whether the prior use of antiplatelet agents may influence DAPT following a TIA. As shown in Table 1, most patients with TIAs were naïve to antiplatelet agents at the time of the acute event. There was no difference in the frequency of DAPT in patients with or without previous ASA use in low- or high-risk categories. Very few patients were on DAPT prior to the presentation. In these patients, the rates of DAPT at discharge were significantly higher when compared to the other two groups. Other factors, including the relationship of DAPT to preexisting vascular risk factors and the ethnicity of the patients, are shown in Table 1.

### 3.3. Distribution of antiplatelet treatment in patients with ischemic stroke

**Figure 2:**
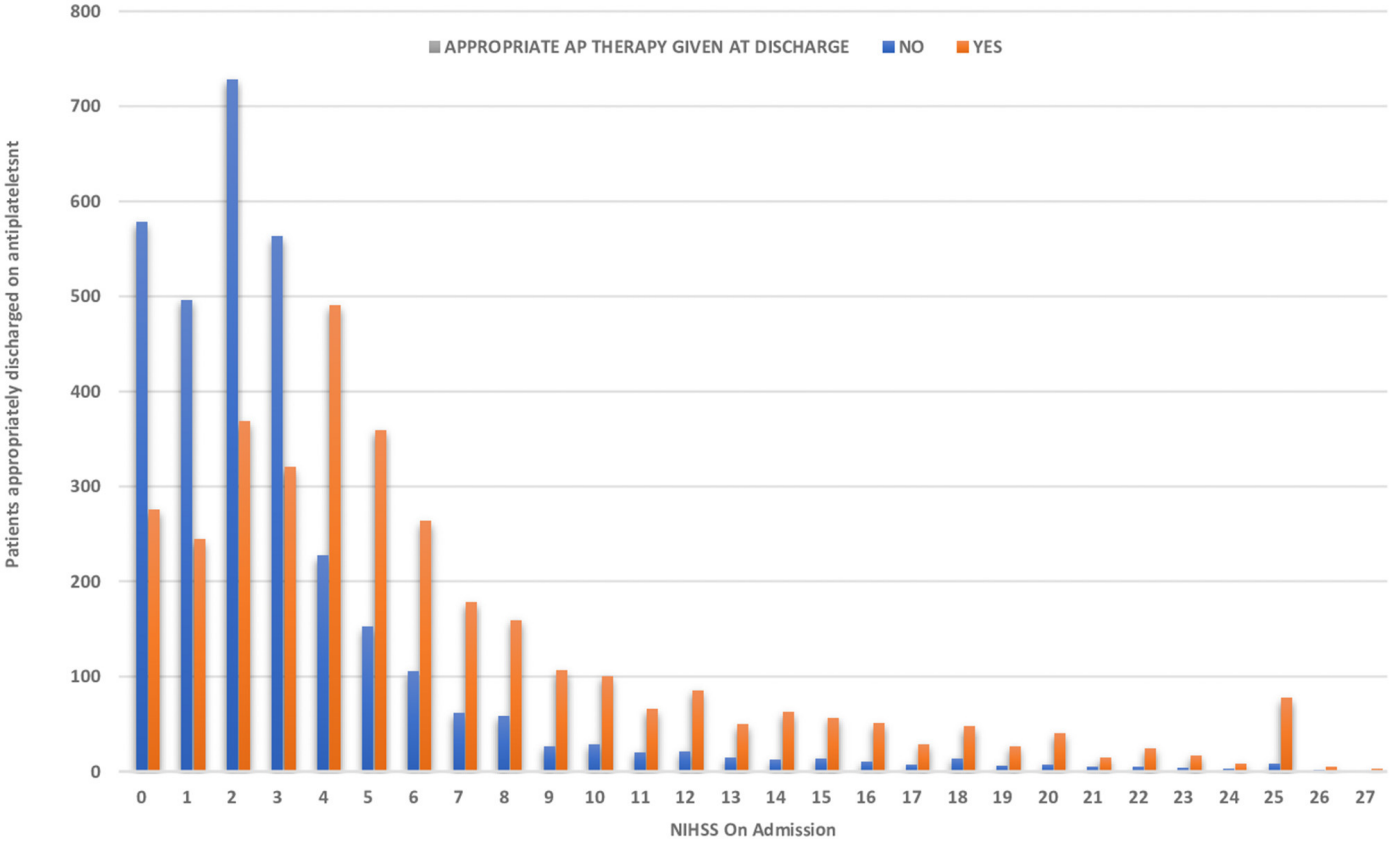
Treatment of DAPT in patients with acute stroke in relationship to the NIHSS.

This group comprised 6,725 patients. As shown in Table 2, 53.1% of patients with acute stroke had an NIHSS score of ≤3. In patients with minor strokes, 1,207/3,572 (33.8%) patients were discharged on DAPT and 2,365/3,572 (66.2%) on SAPT. For patients with an NIHSS of ≥4, SAPT is recommended for antiplatelet therapy by most international guidelines (7, 8, 17, 18). In our patients, SAPT was offered to 2,344/3,153 (74.3%) patients, and DAPT was offered to 809/3,153 (25.7%) patients. As shown in Figure 2, the rate of DAPT use decreased as the NIHSS increased. The rates of DAPT use were similar in patients presenting within <24 h or more than 72 h from the onset of symptoms.

**Table 2:** Baseline characteristics of patients admitted with ischemic stroke and discharged on appropriate therapy.

**Characteristic or investigation**	**All minor strokes (*n* = 3,572)**	**Discharged on inappropriate therapy (*n* = 2,365, 66.2%)**	**Discharged on appropriate therapy (*n* = 1,207, 33.8%)**	***P*-value**	**All major strokes (*n* = 3,153)**	**Not discharged on appropriate therapy (*n* = 809, 25.7%)**	**Discharged on appropriate therapy (2,344, 74.3%)**	***P*-value**
Age, mean, years	54 ± 12.3	54.6 ± 12.7	54.3 ± 11.5	0.43	55.18 ± 37.4	54.1 ± 11.7	55.5 ± 42.9	0.36
Male sex	2,899 (81.2)	1,855 (78.4)	1,044 (86.5)	< 0.001	2,606 (82.7)	702 (86.8)	1,904 (81.2)	< 0.001
Hypertension	2,672 (74.8)	1,744 (65.3)	928 (34.7)	0.04	2,264 (71.8)	620 (27.4)	1,644 (72.6)	< 0.001
Diabetes	2,040 (57.1)	1,311 (64.3)	729 (35.7)	0.005	1,784 (56.6)	487 (27.3)	1,297 (72.7)	0.02
Dyslipidemia	1,778 (49.8)	1,144 (64.3)	634 (35.7)	0.02	1,441 (45.7)	395 (27.4)	1,046 (72.6)	0.04
Prior stroke	374 (10.5)	226 (60.4)	148 (39.6)	0.01	380(12.1)	121 (31.8)	259 (68.2)	0.004
Atrial fibrillation	81 (2.3)	63 (77.8)	18 (22.2)	0.03	133 (4.2)	20 (15.0)	113 (85.0)	0.004
Coronary artery disease	354 (9.9)	190 (53.7)	164 (46.3)	< 0.001	356 (11.3)	123 (34.6)	233 (65.4)	< 0.001
Active smoking	1,048 (29.3)	673 (64.2)	375 (35.8)	0.11	945 (30)	265 (28.0)	680 (72.0)	0.04
Obesity (BMI ≥ 30 kg/m^2^)	919 (26.3)	627 (68.2)	292 (31.8)	0.09	753 (24.8)	172 (22.8)	581 (77.2)	0.02
**Ethnicity**								
Arabs	1,197 (33.5)	812 (67.8)	385 (32.2)	0.29	924 (29.3)	218 (23.6)	706 (76.4)	0.38
South Asian	1,842 (51.6)	1,199 (65.1)	643 (34.9)		1,767 (56)	468 (26.5)	1,299 (73.5)	
Far Eastern	309 (8.7)	197 (63.8)	112 (36.2)		259 (8.2)	73 (28.2)	186 (71.8)	
African	140 (3.9)	97 (69.3)	43 (30.7)		134 (4.2)	31 (23.1)	103 (76.9)	
Caucasian	84 (2.4)	60 (71.4)	24 (28.6)		69 (2.2)	19 (27.5)	50 (72.5)	
**Prior antiplatelet use**								
None	2,808 (78.6)	1,887 (67.2)	921 (32.8)	< 0.02	2,510 (79.6)	615 (24.5)	1,895 (75.5)	< 0.03
Antiplatelet use	764 (21.4)	478 (62.6)	286 (37.4)		643 (20.4)	194 (30.2)	449 (69.8)	
Prior anti-hypertensive use	1,238 (34.7)	851 (68.7)	387 (31.3)	0.02	918 (29.1)	245 (26.7)	673 (73.3)	0.39
Anti-diabetic medication use	1,055 (29.5)	700 (66.4)	355 (33.6)	0.91	738 (23.4)	213 (28.9)	525 (71.1)	0.02
Prior statin use	829 (23.2)	540 (65.1)	289 (34.9)	0.45	659 (20.9)	184 (27.9)	475 (72.1)	0.13
Initiation of antiplatelet within 24 h	1,610 (45.1)	985 (61.2)	625 (38.8)	< 0.001	1,386 (44.0)	387 (27.9)	999 (72.1)	< 0.01
Mean duration to start antiplatelet (h)	13.5 ± 17.7	14.2 ± 20.3	12.03 ± 11.2		16.8 ± 20.9	14.9 ± 22.6	17.5 ± 20.2	0.003
**Antiplatelet given from onset**								
< 24 h	1,610 (45.1)	985 (61.2)	625 (38.8)	< 0.001	1,386 (44)	387 (27.9)	999 (72.1)	< 0.001
Between 24 and 48 h	882 (24.7)	616 (69.8)	266 (30.2)		1,179 (37.4)	259 (22.0)	920 (78.0)	
Between 48 and 72 h	592 (16.6)	391 (66.0)	201 (34.0)		362 (11.5)	118 (32.6)	244 (67.4)	
>72 h	488 (13.7)	373 (76.4)	115 (23.6)		226 (7.2)	45 (19.9)	181 (80.1)	
NIHSS on admission (mean)	1.5 ± 1.1	1.54 ± 1.1	1.61 ± 1.1	0.08	8.61 ± 5.5	7.50 ± 4.6	9 ± 5.7	< 0.001
**NIHSS admission**								
0	853 (23.9)	578 (67.8)	275 (32.2)	0.32				
1	740 (20.7)	496 (67.0)	244 (33.0)					
2	1,096 (30.7)	728 (66.4)	368 (33.6)					
3	883 (24.7)	563 (63.8)	320 (36.2)					
4					717 (31.0)	227 (31.7)	490 (68.3)	0.04
5					511 (22.1)	152 (29.7)	359 (70.3)	
6					368 (15.9)	105 (28.5)	263 (71.5)	
7					239 (10.3)	61 (25.5)	178 (74.5)	
8					217 (9.4)	58 (26.7)	159 (73.3)	
9					132 (5.7)	26 (19.7)	106 (80.3)	
10					128 (5.5)	28 (21.9)	100 (78.1)	
**Toast classification**								
Small vessel disease	2,126 (59.6)	1,405 (66.1)	721 (33.9)	< 0.001	1,308 (41.6)	327 (25.0)	981 (75.0)	< 0.001
Large vessel disease	575 (16.1)	329 (57.2)	246 (42.8)		848 (26.9)	279 (32.9)	569 (67.1)	
Cardioembolic	478 (13.4)	353 (73.8)	125 (26.2)		571 (18.1)	121 (21.2)	450 (78.8)	
Stroke of determined origin	207 (5.8)	134 (64.7)	73 (35.3)		278 (8.8)	60 (21.6)	218 (78.4)	
Stroke of undetermined origin	179 (5.0)	138 (77.1)	41 (22.9)		143 (4.5)	21 (14.7)	122 (85.3)	

DAPT, dual antiplatelet therapy; IV, intravenous; NIHSS, National Institute of Health Stroke Scale; RBS, random blood sugar; HDL, high density lipoprotein; LDL, low density lipoprotein; BMI, body mass index; mRS, modified Rankin score; CABG, coronary artery bypass Graft; PCI, percutaneous coronary intervention; MACE, major cardiac adverse event.

Independent sample t-test was applied to test for significant differences across antiplatelet use for all continuous variables, whereas the chi-square test was used for all categorical variables.

The relationship between vascular risk factors and the rates of DAPT used is shown in Table 2. There were no differences in the use of DAPT in relation to the prior use of antihypertensive, antidiabetic, and statin medications in patients with acute stroke.

We used the TOAST classification for the underlying diagnosis of acute stroke. As shown in Table 2, DAPT use was the highest in minor strokes related to large vessel disease. DAPT was used in 246 out of 575 patients (42.8%) in this high-risk group.

### 3.4. Trends in DAPT use

We next explored some potential reasons for the low use of DAPT in our cohort. We initially evaluated whether there was a difference in the use of antiplatelet agents among the stroke consultants directly responsible for the care of the patients. In all, there were 13 physicians who were responsible for the care of stroke patients. There were no differences seen in the appropriate or inappropriate use of DAPT across the physician group (data not shown). Second, we evaluated the treatment trends across the years of the study (2014–2022) and in the periods before the CHANCE study (published in 2014) and following the publication of the CHANCE and POINT study (published in 2018), as well as in the post-POINT time period. We were not able to show any significant increase in DAPT use over time or following the publication of the landmark studies (data not shown).

The results of the analysis on patients with high-risk TIAs and low-risk TIAs and in patients with minor and non-minor stroke patients comparing their discharged status on appropriate and inappropriate DAPT are shown in Supplementary Tables 1, 2.

## 4. Discussion

In patients with TIAs and acute stroke, most guidelines and expert opinions recommend early initiation of antithrombotic medications (7, 8, 17, 18, 31, 32). An early initiation of DAPT within 24 h of the onset of symptoms in patients with high-risk TIAs and minor stroke has been shown to be superior to SAPT in preventing early recurrent stroke (15, 16, 32, 33), with a slightly higher risk of hemorrhagic complications (34). Furthermore, a recent sub-analysis of the CHANCE and POINT studies has shown that the risk of recurrent stroke is highest in the initial 3 days and later decreases rapidly (35, 36). Research determining if clinical practice has changed following the publication of the guidelines is important. Data from the Get-With-The-Guidelines have shown that only 47% of patients with minor strokes were being offered DAPT (19, 20). There was also an inappropriate 42.6% use of DAPT for patients with non-minor strokes (19, 20). To the best of our knowledge, there are no studies on the appropriate use of DAPT in patients with stroke from other regions of the world. Similar studies on the appropriateness of antiplatelet medications in patients with TIAs have also not been published.

There are several important features in our analysis. Our study shows that DAPT continues to be underutilized in patients presenting early with high-risk TIAs and minor strokes. Only 42.3% of patients with TIAs and 33.8% of patients with minor strokes in our cohort were treated appropriately with DAPT at the time of discharge. DAPT was inappropriately used in 34.4% of low-risk TIAs and 25.7 % of non-minor stroke patients. These numbers are similar to the report from the United States (19, 20). Additionally, there was no increase in the rates of DAPT following the release of the POINT trial results or the publication of the revised guidelines. We also did not show any increase in the use of DAPT in patients who were on ASA at the time of the new event or in patients with higher vascular risk factors.

Rapid identification of patients at high risk for recurrence is the most important initial step prior to the initiation of appropriate antithrombotic treatment. The ABCD2 score is perhaps the most widely used score to identify high-risk TIA patients (13). A score of ≥ 4 is considered “high-risk” and has been used to enter patients into clinical trials (15, 16). In a recent meta-analysis of 29 studies, the 7-day risk of stroke was 10.2% in patients with an ABCD2 score of ≥ 4. A significantly lower risk of stroke of 3.2% was evident with lower ABCD2 scores (37). The meta-analysis also showed that while the higher scores were sensitive but not very specific for identifying patients at high early stroke risk (13), the addition of imaging to the ABCD2 score may improve its specificity (38).

There are additional clues from the recent large trials that identify TIA and minor strokes where DAPT is highly effective. In the CHANCE study, most strokes in both arms of the study occurred within the first 2 weeks of the symptom onset (39). Similarly, the POINT study also showed that recurrent stroke was most common in the initial 3 days, and DAPT was likely to be effective if initiated within 3 days from the onset of symptoms (40). Additionally, in both the POINT and CHANCE trials, patients with high blood pressure at the time of enrollment received a greater benefit from DAPT (36, 41). The CHANCE trial also showed that increasing NIHSS, poor control of hypertension during follow-up, a lack of use of lipid-lowering agents, and the presence of intracranial atherosclerosis were additional risk factors for a higher risk of stroke recurrence (41).

The NIHSS score is used to define patients with minor strokes for stroke prevention trials. An NIHSS score of **≤**4 was used for the identification of minor strokes in the two recent large DAPT studies (15, 16). This arbitrary separation of a minor from a non-minor stroke is problematic, as there are no biological reasons to expect a difference in the outcome. It is, therefore, understandable that there is no clear separation of the use of DAPT and SAPT in patients when the NIHSS score increases beyond 4. Our results in a large cohort show that DAPT use gradually decreases as the NIHSS rises but remains high with an NIHSS of 10. Almost 30% of patients were prescribed DAPT in the 5–10 NIHSS range. We do not know if the risk of complications from DAPT rises with a high NIHSS. In a small study of 119 patients with stroke and TIAs, bleeding risk increased with increasing NIHSS, but this did not lead to a higher mortality rate (42). Another study presented at the American Academy of Neurology reviewed their results in 1,316 patients in a prospectively collected database. The risk of cerebral hemorrhage was related to the size of the cerebral infarction and not to the single or dual antiplatelet treatment (43). Kim et al. presented their data from a larger national registry in South Korea that included 4,461 patients with moderate-to-large strokes (NIHSS 44-15). In total, 52% of patients were treated with SAPT, and 47% received DAPT. During a 3-month follow-up, there were no significant differences in the recurrent stroke rates, but the mortality was significantly lower in the DAPT group. The bleeding risk with the two therapies was, however, not reported (44). The limited available evidence suggests that DAPT is likely safe for patients with non-minor strokes.

We were not able to determine the reasons for the low use of DAPT in high-risk TIA patients. The prescribing patterns were similar for all the consultant neurologists. We did not see any increase in the use of DAPT with the availability of the POINT study or the wide availability of the revised guidelines. The emergency department is usually the first point of contact for the patient to be managed by the medical team. In a recent survey on the use of DAPT by emergency physicians in the United States, 68% of participants reported not using clinical prediction rules, and only 18% reported using the ABCD2 score to inform treatment choices (45). Nearly half of the surveyed physicians reported that they would use ASA, and more alarmingly, only 2% reported that they would treat high-risk TIA or minor strokes with DAPT (45). These are somber results and an opportunity for improving early emergency care for patients at the highest risk for recurrence.

The underutilization of DAPT in high-risk patients is difficult to understand. An important consideration is the perceived higher risk of complications with DAPT use. The risk of bleeding with short-term DAPT is generally low. In the POINT trial, the risk of major hemorrhage was 0.2 % in the SAPT arm and 0.9% in the DAPT (46). In our study, the use of DAPT was similar in consultants with or without additional stroke fellowship training and did not improve over time. The Get-With-The-Guidelines program, however, showed remarkable differences between DAPT use in comprehensive centers (55.2%) vs. primary stroke centers (22.3%) in the management of minor strokes (20). Other factors that contribute to the lower use of DAPT include the occurrence of a stroke while the patient is already on ASA (47), allergic to ASA (48), recent hemorrhage while on ASA (49), and perceived or known drug resistance to ASA or clopidogrel (49). It is not uncommon to have a vascular event while on ASA. In our series, approximately 20% of patients were on ASA at the time of their TIA or stroke. The recommended approach is to add clopidogrel instead of switching to another agent (49). Allergic reactions to ASA are rare, and investigating for resistance is generally not recommended (49). Pseudo-resistance to ASA may be more common and may be related to enteric coating (49).

There are limitations to our study. Although the data were entered prospectively, this is a retrospective analysis and does not document the reasons DAPT or SAPT were chosen for individual patients. Although we recorded a large number of variables, we did not record specific complications from individual medications; therefore, we cannot document bleeding outcomes for the patients. We specifically did not look for symptomatic intracranial stenosis patients in whom arguably the use of dual antiplatelets for 90 days or longer is recommended (50). This study is limited to the use of antiplatelet medications in a large comprehensive stroke program in the Middle East, and the data may not be generalizable to other populations. The strength of the study is that the data were collected in a prospective registry at a comprehensive stroke center in a multi-ethnic population from the Middle East.

In summary, we reviewed the antiplatelet treatment patterns of TIAs and strokes during the last 8 years. Important observations include that there is low use of DAPT in high-risk TIA and minor stroke patients in an ethnically diverse population from the Middle East and Southeast Asia. This low uptake of DAPT has not improved over time, despite the publication of revised guidelines. More studies on enhancing evidence-based adaptation of DAPT in the appropriate patients should be targeted for quality improvement initiatives.

## Data availability statement

The original contributions presented in the study are included in the article/Supplementary material, further inquiries can be directed to the corresponding author.

## Ethics statement

The studies involving humans were approved by Medical Research Centre of Hamad Medical Corporation. The studies were conducted in accordance with the local legislation and institutional requirements. The Ethics Committee/Institutional Review Board waived the requirement of written informed consent for participation from the participants or the participants' legal guardians/next of kin because this is Registry Study and appropriate data approved was prospectively collected from the database.

## Author contributions

HN: Writing—original draft, Formal analysis, Methodology. NA: Formal analysis, Methodology, Supervision, Writing—original draft. SA-J: Methodology, Validation, Writing—review & editing. RU: Data curation, Methodology, Writing—review & editing. SJ: Data curation, Methodology, Writing—review & editing. DM: Data curation, Methodology, Writing—review & editing. BB: Data curation, Methodology, Writing—review & editing. SS: Methodology, Writing—review & editing, Data curation. AS: Writing—original draft, Conceptualization, Writing—review & editing.
