# Supplementary material for: Appropriate use of antiplatelet medications following transient ischemic attacks and stroke: a 9-year study from the Middle East
Source: Front Neurol. 2023 Nov 9;14:1269292. doi: 10.3389/fneur.2023.1269292 (PMC10666165; doi:10.3389/fneur.2023.1269292)
Supplement: Supplementary file 1 [file Data_Sheet_1.pdf]

Supplementary Table 1- Univariate short-term 90- days outcome and major cardiovascular events (MACE) in patients admitted with TIA and discharged on appropriate versus inappropriate dual antiplatelet therapy (DAPT). Significantly more "high-risk" TIA patients discharged on "appropriate" DAPT had a mRS of 0-2 at 90 days. MACE at one year was no different in the patients on appropriate and inappropriate treatment in both the high-risk and low-risk TIA patients.

Characteristic or Investigation	Total High Risk TIA's (n=666)	Discharged on Inappropriate therapy (n=384, 57.7%)	Discharged on Appropriate Therapy (n= 282, 42.3%)	P-Value	Total Low Risk TIA's (n= 691)	Discharged on Inappropriate therapy (n=238, 34.4%)	Discharged on Appropriate Therapy (453, 65.6%)	P-Value
**Prognosis – At Discharge**								
Good (mRS 0-2)	616 (92.5)	348 (90.6)	268 (95)	0.03	661 (95.7)	233 (97.9)	428 (94.5)	0.04
Poor (mRS 3-6)	50 (7.5)	36 (9.4)	14 (5)		30 (4.3)	5 (2.1)	25 (5.5)	
**Prognosis – 90-Days**								
Good (mRS 0-2)	514 (92.1)	277(88.5)	237 (96.7)	<0.001	586 (95)	212 (95.9)	374 (94.4)	0.42
Poor (mRS 3-6)	44 (7.9)	36 (11.5)	8 (3.3)		31 (5)	9 (4.1)	22 (5.6)	
**MACE At One Year**								
Recurrent stroke	13 (2.0)	6 (46.2)	7 (53.8)	0.39	7 (1.0)	4 (57.1)	3 (42.9)	0.20
Post Stroke MI	5 (0.8)	5 (100.0)	0	0.05	5 (0.7)	2 (40.0)	3 (60.0)	0.79
All Mortality	8 (1.2)	6 (75.0)	2 (25.0)	0.32	3 (0.4)	1 (33.3)	2 (66.7)	0.97
Cardiac arrest post stroke	1 (0.2)	0	1 (0.4)	0.24	-	-	-	-
Post stroke (CABG or PCI)	4 (0.6)	4 (100.0)	0	0.09	2 (0.3)	0	2 (100.0)	0.30
Total MACE at 1 year	27 (4.1)	18 (66.7)	9 (33.3)	0.33	14 (2.0)	6 (42.9)	8 (57.1)	0.50

DAPT- Dual Antiplatelet Therapy, IV- intravenous, mRS- Modified Rankin Score, CABG- Coronary artery bypass Graft, PCI- Percutaneous Coronary Intervention, MACE- Major Cardiovascular Adverse Event

Supplementary Table 2- Univariate short-term 90-days outcome and one year major cardiovascular (MACE) in minor and non-minor stroke patients discharged on appropriate and inappropriate DAPT. The short-term and one year MACE was significantly better in patients with appropriate DAPT.

Characteristic or Investigation	All Minor Strokes (n= 3572)	Not Discharged on Appropriate therapy (n= 2365, 66.2%)	Discharged on Appropriate Therapy (n= 1207, 33.8%)	P-Value	All non-minor Strokes (n= 3153)	Not Discharged on Appropriate therapy (n= 809, 25.7%)	Discharged on Appropriate Therapy (2344, 74.3%)	P-Value
**Prognosis – At Discharge**								
Good (mRS 0-2)	2869 (80.3)	1833 (77.5)	1036 (85.8)	<0.001	1261 (40)	409 (50.6)	852 (36.3)	<0.001
Poor (mRS 3-6)	703 (19.7)	532 (22.5)	171 (14.2)		1892 (60)	400 (49.4)	1492(63.7)	
**Prognosis – At 90-Days**								
Good (mRS 0-2)	2249 (85.4)	1405(83.2)	844 (89.3)	<0.001	1223 (56.1)	383 (68)	840 (51.9)	<0.001
Poor (mRS 3-6)	384 (14.6)	283 (16.8)	101 (10.7)		957 (43.9)	180 (32)	777 (48.1)	
**MACE At One Year**								
Recurrent stroke	58 (1.6)	34 (58.6)	24 (41.4)	0.23	50 (1.6)	21 (42.0)	29 (58.0)	0.008
Post Stroke MI	22 (0.6)	8 (36.4)	14 (63.6)	0.003	14 (0.4)	6 (42.9)	8 (57.1)	0.14
All Mortality	44 (1.2)	36 (81.8)	8 (18.2)	0.03	134 (4.2)	30 (22.4)	104 (77.6)	0.37
Cardiac arrest post stroke	8 (0.2)	6 (75.0)	2 (25.0)	0.59	24 (0.8)	7 (29.2)	7 (70.8)	0.69
Post stroke (CABG or PCI)	19 (0.5)	6 (31.6)	13 (68.4)	0.001	18 (0.6)	8 (44.4)	10 (55.6)	0.06
Total MACE at 1 year	129 (3.6)	78 (60.5)	51 (39.5)	0.16	202 (6.4)	58 (28.7)	144 (71.3)	0.30

DAPT- Dual Antiplatelet Therapy, IV- intravenous, mRS- Modified Rankin Score, CABG- Coronary artery bypass Graft, PCI- Percutaneous Coronary Intervention, MACE- Major Cardiovascular Adverse Event
